# Consenting for themselves: a qualitative study exploring a Gillick Competence assessment to enable adolescents to self-consent to low-risk online research

**DOI:** 10.1136/bmjopen-2024-090747

**Published:** 2025-03-04

**Authors:** Maria Loades, Lara Willis, Emma Wilson, Grace Perry, Melanie Luximon, Christy T C Chiu, Nina Higson-Sweeney

**Affiliations:** 1 Department of Psychology, University of Bath, Bath, UK

**Keywords:** Child, Adolescent, Parents, QUALITATIVE RESEARCH, Research Design

## Abstract

**Abstract:**

**Background:**

Providing digital mental health interventions online could expand access to help for young people, but requiring parental consent may be a barrier to participation. We therefore need a method that enables young people <16 years old (ie, presumed competent in the UK) to demonstrate Gillick Competence (understanding of purpose, process, potential benefits and potential harms) to self-consent to online, anonymous, low-risk studies.

**Aim:**

To explore whether a new method for assessing Gillick Competence to participate in low-risk, anonymous online studies is acceptable to both young people and parents.

**Methods:**

We interviewed 15 young people aged 13–5 years and 12 parents of this age group in the UK. Using a qualitative approach, we explored the acceptability of a series of multiple-choice questions (MCQs) designed to assess understanding of a specific online self-help research study testing a self-kindness intervention.

**Results:**

The MCQ answers that participants gave mostly corresponded with their narrative explanations of their understanding during interviews. Young people and parents thought that the process was empowering and could increase access to research while also promoting independence. However, they emphasised the importance of individual differences and different research contexts and highlighted the need for safeguards to be in place.

**Conclusions:**

The MCQs were acceptable to both young people and parents, providing preliminary evidence for the potential of this process for allowing <16s to self-consent to online, anonymous, low-risk mental health research. Further research is needed to validate the effectiveness of this process among a diverse range of populations and research contexts.

**STRENGTHS AND LIMITATIONS OF THIS STUDY**

- We involved young people in the development of the multiple-choice questions.
- We embedded questions about the process within a study of an intervention in which the process would be used, meaning respondents had a concrete example and were the potential end users.
- We also used a real study information sheet as part of the materials for the interviews.
- However, this meant that participants consented to the interviews and then had to answer questions about a different study information sheet, which may have been confusing.
- Ethical requirements meant that we could only include young people who were able to secure parental consent to take part.

## Introduction

 In the context of research, consent refers to the process of an individual providing their agreement to take part in a study. At the outset of any study and prior to data collection, researchers must ensure that potential participants are fully informed about what a study will involve and obtain their voluntary consent to participate.^1^ For consent to be considered valid, the consenting individual needs to be competent to make that decision.^2^ The legal position, which does vary from country to country, tends to be that competence to consent to take part in research studies is assumed in adults, whereas in minors (ie, anyone under the legal age of adulthood), competence is not presumed.^3^ Therefore, consent is commonly sought from an individual with parental or caring responsibilities (henceforth ‘parents’). Yet, some under 16s may be competent to make the decision themselves, and in the UK, the legal position allows for this if competence to consent can be proven.^4^ Providing researchers have direct, interactive contact with potential participants, researchers can assess this when they first meet with a young person. However, this may not be possible in studies that are conducted online, particularly where it is important to maintain anonymity. It is therefore important to develop a method that allows young people (YP) to demonstrate their competence to consent for use within online research.

From a legal perspective in the UK, YP who are at least 16 years old are presumed to be competent to consent; however, those <16 may also be considered able to make decisions and consent for themselves if they can demonstrate Gillick Competence (GC). GC was primarily established so that YP can consent to medical treatments. GC refers to whether a child has a ‘sufficient understanding and intelligence to enable them to understand fully what is proposed’.^4^ Available evidence indicates that the ability to understand medical decisions among YP who are ≥13 years old is similar to that of adults,^5^ although there is considerable individual variability in cognitive, socio-emotional and neurological development,^6^,^8^ and younger children have demonstrated GC in some instances.^9 10^ In the context of psychological therapy, in UK clinical practice, GC is commonly used for YP consenting to receive treatment,^11^ particularly at the stage of early mental health help like counselling provided in contexts like schools.

In consenting to take part in research studies, GC means that YP can understand the purpose of the study, what taking part will involve and the potential risks and benefits of participation.^4 12^ Consistent with this, UK research ethical committee guidance allows for <16s to consent to participate in research for themselves, providing they can demonstrate GC.^13^ However, it can be difficult to assess competence to consent online, so these practices are not typically followed in online, anonymous studies, which tend to require YP <16 years of age who want to take part to seek parental consent.^14^ But obtaining parental consent online is both impractical and logistically difficult to ensure as it is nearly impossible to determine who completed an online, anonymous consent form.^15^

Requiring parental consent for minors can create several barriers to research participation. For example, YP may be reluctant to participate in studies about sensitive topics (eg, mental health, LGBTQIA+ identity) which their parents are unaware of.^16 17^ This could lead to lower response rates and smaller samples, which could affect study power and the ability of intervention research to detect treatment effects.^18^ Furthermore, some YP (eg, those who belong to marginalised groups or have a conflictual relationship with their parents) may be systematically less likely to participate, which impacts the representativeness of a study’s findings.^19^ For instance, given that parent-teen conflict is a known risk factor for developing depression,^20^ YP who are at greater risk of depression and poor mental health may also be those who are least likely to feel able to ask their parents for permission to take part in mental health research. Hence, requiring parental consent can not only reduce representation and generalisability of findings but can also impact the validity and reliability of the consent process. It also potentially violates YP’s rights to engage in research afforded to them in the United Nations Convention on the Rights of the Child as well as by the Children’s Act of 1989 in England and Wales. Furthermore, consent has been highlighted by researchers and ethics committee members as a major challenge faced in conducting child and adolescent research.^21^ Having a method by which GC can be assessed in online, anonymous, low-risk research studies therefore offers a potentially more inclusive and accessible alternative to parental consent.

Although the option of GC assessment to consent to research has yet to become common practice within online studies conducted in the UK, an approach has been developed in Australia that allows minors to consent to online research participation through GC, which is assessed using a multiple-choice test which they complete before the consent form.^22^ This was used in a recent study that examined the impact of COVID-19 on the lives and mental health of Australian adolescents (aged 12–18) via online surveys,^23^ where all potential participants were required to pass a GC assessment task before consenting to participate.

In summary, there are many benefits to offering the option of a GC assessment process to YP who wish to participate in online research studies, including improved data quality, broader generalisability of findings, increased inclusivity and empowerment of YP to be in control of their research involvement. Drawing on the approach used to assess GC in online, anonymous research studies conducted in Australia, we aimed to develop and evaluate a process for assessing GC within online, anonymous, low-risk mental health intervention studies in a UK context. Specifically, we focused on exploring its acceptability to two key stakeholder groups: YP, as it is vital for them to be actively engaged in shaping the future of research designed for them,^24^ and parents, who play a vital role within the process of consent, yet rarely have their perspectives considered in research on this topic.^25 26^

## Methods

### Design

Based on the Australian GC assessment model,^23^ with additional input from YP in the UK, we developed a process for assessing GC in low-risk, online survey studies using multiple-choice questions (MCQs) to assess comprehension of the study information (ie, the purpose and process) and implications (ie, risks and benefits) of taking part in the study. We then conducted a qualitative study embedded within a single-arm pre-post intervention programme evaluation of a single-session self-help online intervention, Project Care UK. At the information sheet stage of Project Care UK, YP aged 13–15 were invited to opt into an additional qualitative study to help us assess the feasibility and acceptability of this new process for future use in online studies. Specifically, our research questions were:

1. Do YP’s understanding of the study information match the way they respond to the MCQs developed for the GC assessment?
2. What do YP think about the GC assessment process and the MCQs?

We also recruited a sample of parents of YP aged 13–15 to explore their thoughts regarding the GC assessment process and our MCQs.

### Participants

Any UK-based YP aged 13–15 who considered taking part in Project Care UK by viewing the information sheet were also offered the opportunity to volunteer to take part in an additional qualitative study. Opting in to the qualitative study did not depend on them consenting to take part in Project Care UK.

Separately, any UK-based parents of YP aged 13–15 (not necessarily linked to a participating YP) could sign up to participate in the qualitative study. YP and parent interviews were conducted independently, with data remaining unpaired even if a parent-child dyad participated.

Based on the sample size we anticipated would provide sufficient information power to answer our research questions,^27^ we aimed to recruit at least 10 YP and 10 parents.

### Materials

The GC assessment MCQs were developed specifically for this study, based on prior research in Australia.^23^ Initially, MLo drafted four MCQs, each of which had four answer options, one of which was correct. The MCQs assessed (1) the understanding of what the study involved, (2) why it was being done, (3) what the risks were of taking part and (4) the potential benefits of taking part (see table 1). MLo shared the draft MCQs with a colleague who was chair of the psychology department’s ethics committee. The colleague provided feedback and suggestions regarding simplifications. MLo made these amendments and then sought input from two young adults (undergraduate students), resulting in several further changes to the wording of the questions and answer options provided. The young adults then reviewed the changes and discussed and agreed refinements in a meeting with MLo.

**Table 1:** Gillick Competence multiple-choice questions

Aspect of Gillick Competence	Question asked	Multiple-choice questions options
Purpose	What is this study about?	We are testing a 10-session self-help intervention.
		**We are testing an online self-help single session intervention**.
		We are testing a single session intervention in which you will talk to a therapist in person.
		We are testing a 6-session therapist-delivered intervention.
Process	What will you be asked to do?	I will be asked to do an activity online but will not be asked to answer questions.
		I will be asked questions but will not do any activities.
		I will be asked to do an activity online only.
		**I will be asked to answer some questions online, do an activity, and then answer some more questions online**.
Benefits	How could this help you?	**Doing this study could teach me new ideas about ways I could help myself and be kinder to myself**.
		Doing this study could teach me ways to pass my exams.
		Doing this study could teach me about using a computer.
		Doing this study has no potential benefits to me.
Harms	What are the risks of taking part?	I will have to share private information like my name, which will be shared publicly.
		I will have to talk to someone about my feelings.
		**Some questions could be upsetting**.
		There are no risks.

**Responses in bold indicate correct response.**

### Procedure

Project Care UK was advertised on social media platforms (eg, Instagram, X - formerly Twitter, Threads) and via mailing lists and relevant charities. We paid (<£20) to boost the study advert on Instagram for a few days during August 2023, specifically targeting UK-based YP in the relevant age range. Interested participants used a link/QR code to access a secure screening platform on Qualtrics to determine eligibility. Once potential participants passed the eligibility checks (self-reporting that they were aged 13–18 and living in the UK), they were provided with an information sheet about Project Care UK. Before they were presented with the consent processes for Project Care UK, those who were aged 13–15 were informed about the current study and given a link to a separate Qualtrics project where they could access the study information sheet, consent form and leave their contact details for the current study (July to October 2023). YP interested in taking part were asked to have their parent complete the written consent process for the YP to take part in the current study and to select a pseudonym which would be attached to their anonymous data (pseudonyms included in the ‘Results’ section). Note that Project Care UK findings will be reported elsewhere and the protocol can be accessed via the Open Science Framework (https://osf.io/cje2w/)

We also used convenience sampling to advertise the qualitative study directly on social media and via mailing lists, mainly aiming our adverts at parents. Following the link/QR code on the advert routed potential participants to the Qualtrics project for the current study which included detailed information about the qualitative study, an online consent form and space to provide their contact details.

Qualtrics responses were screened for potential bots (ie, filtering out Qualtrics forms which scored ≤0.5 on the RECAPTCHA question as this indicates that the respondent might not be genuine). Next, all ineligible responses (ie, those who did not meet the study inclusion criteria) and incomplete forms were filtered out. Members of the research team (EW, LW) conducted further manual screening checks on potential participants whose contact details seemed suspicious, following a standard operating procedure developed by our team (available from the corresponding author on request and based on refs.^28 29^). The research team (EW, LW) then contacted participants who passed these checks via telephone or email to arrange an interview.

Semistructured interviews incorporating think-aloud techniques^30^ were conducted, recorded and transcribed on Microsoft Teams. In think-aloud interviews, participants are asked to talk out loud while completing a certain task^31^ with interviewers using pre-prepared prompts to encourage them to verbalise their thoughts and feelings on content, design and usability.^32^ Consent was verbally reconfirmed at the beginning of the interviews. Interviews were conducted by EW (DClinPsy) and LW (DClinPsy), both of whom were trainee clinical psychologists at the time. EW and LW had received training in qualitative research as part of their clinical training and were working under the supervision of an experienced clinical psychologist (MLo). EW or LW did not establish relationships with participants prior to conducting the interviews; however, they were responsible for contacting participants via telephone and email to arrange interviews, so both had superficial contact with participants before the interviews.

At the beginning of the interviews, EW and LW introduced themselves (ie, who they were and their occupation) to participants, but they did not detail their research goals, interests or expectations within this. During the interview, YP participants were asked to read the Project Care UK information sheet and complete the GC assessment MCQs independently, verbalising each answer. Participants were then asked a series of questions (see online supplemental material S1 for interview schedule) exploring their perspectives on the process of consent, parental consent versus consenting for themselves and their understanding of the information provided about Project Care UK within the information sheet. This included checking their understanding of the four aspects of GC (what, why, risks, benefits). Finally, the interviewer shared the MCQs one at a time on their screen using a series of Microsoft PowerPoint slides and asked the participant to think-aloud about each one. Parents were asked very similar questions, although they did not complete the GC MCQs (see online supplemental material S3 for interview schedule).

During the first few YP interviews we conducted, we noticed that participants were getting confused about which study we were asking about (ie, qualitative study or the Project Care UK study). As a result, we changed the sequence of questions on the interview schedule after the eighth interview. From this point onwards, participants were asked about their perspectives on the topic of parental consent versus GC before completing the GC MCQs and then undertaking the think-aloud task regarding the content, design and usability of the GC MCQs (see online supplemental material S2 for revised interview schedule). No repeat interviews were completed for those participants who completed interviews prior to this change.

Interviews lasted approximately 45 min, and participants who completed a qualitative interview received a £10 voucher as a token of appreciation and were signposted to sources of support as part of the debrief procedure. Transcriptions created by Microsoft Teams were checked and corrected verbatim for accuracy by supervised undergraduate students; these were not returned to participants prior to data analysis.

The semistructured interview was piloted with a YP (aged 16) to provide feedback, who advised the researchers to spend more time explaining what the research involved, which was subsequently incorporated into the interview schedule. No further changes were made at this stage to the flow of the interview schedule as the pilot interview seemed to flow well. This pilot interview was not treated as generating data for the current study.

### Patient and public involvement

YP advisors highlighted the need for this study, contributing to its conceptualisation. YP advisors, both undergraduate students (young adults), co-developed the MCQs for the GC assessment process, one of whom is a co-author on the current study. A YP advisor (early adolescent, aged between 12 and 17) also reviewed the study documentation at the planning stage and helped us pilot the interview procedures for the current study.

### Analysis

We used reflexive thematic analysis^33^,^35^ from a contextualist, critical realist position which recognises an external reality that is not separable from the context in how it manifests or is understood.^36^ The data were analysed by the research team, and the findings were not shared with the participants. The parent and YP data were analysed separately (parent data analysis led by EW; see Wilson (2024) DClinPsy thesis; YP data analysis led by LW; see Willis (2024) DClinPsy thesis; these full separate reports are available through theses in the University of Bath library repository), with input from MLo and NH-S, who then synthesised and integrated the thematic analyses for this article. Reflexivity was maintained through regular supervisory conversations, and reflexive logs kept by EW and LW during data collection and analysis. We acknowledge that all the authors are female by birth and have psychology careers, although ranging from undergraduate to mid-career. Most are white British. One author (MLo) is a parent.

For face validity purposes, we also used descriptive statistics (counts, frequencies) to compare YP answers to the MCQs given at the beginning of the interviews to their narrative descriptions of their understanding of each of the components of the study assessed by the MCQs.

## Results

We conducted 15 interviews with YP aged 13–15 and 12 interviews with parents, resulting in an overall sample of 27 participants (see table 2 for demographics). One YP participant consented to participate but withdrew prior to being interviewed for personal reasons.

**Table 2:** Demographics of participants in qualitative substudy

Characteristic	Young people participants (n=15)	Parent participants* (n=12)
*Age (years)*		
13	3	3
14	7	7
15	5	2
*Sex at birth*		
Male	10	4
Female	5	8
*Ethnicity*		
White British	11	10
Asian/Asian British	2	1
Black/Black British	1	
Mixed	1	1

* Parent demographics refer to the characteristics of their adolescent child.

### Narrative understanding of GC MCQs

As shown in table 3, YP participants’ performance on the MCQ tended to be consistent with their narrative explanations (11 participants, 73.3%). However, four participants answered the MCQs correctly but showed limited understanding in response to at least one open-ended question. This tended to be the first question (about why the study is being conducted) as there appeared to be confusion regarding whether the open-ended questions were asked in relation to the GC substudy or Project Care UK. In later interviews, when we provided a clearer reminder that these MCQs were referring to the Project Care UK study information, participants’ responses were better matched.

**Table 3:** Frequencies with which participants demonstrated GC comparing MCQ responses to narrative understanding

	Demonstrated narrative understanding of all four GC aspects	Did not demonstrate narrative understanding of at least one GC aspect	Total
All MCQs correct	3* (20%)	4 (26.67%)	7 (46.67%)
At least one MCQ wrong	0 (0%)	8* (53.33%)	8 (55.33%)
Total	3 (20%)	12 (80%)	15

Numbers are frequencies (percentage of total sample).

* Young people whose responses from the MCQs matched their narrative understanding.

GC, Gillick Competence; MCQs, multiple-choice questions

### Thematic analysis

**Figure 1:**
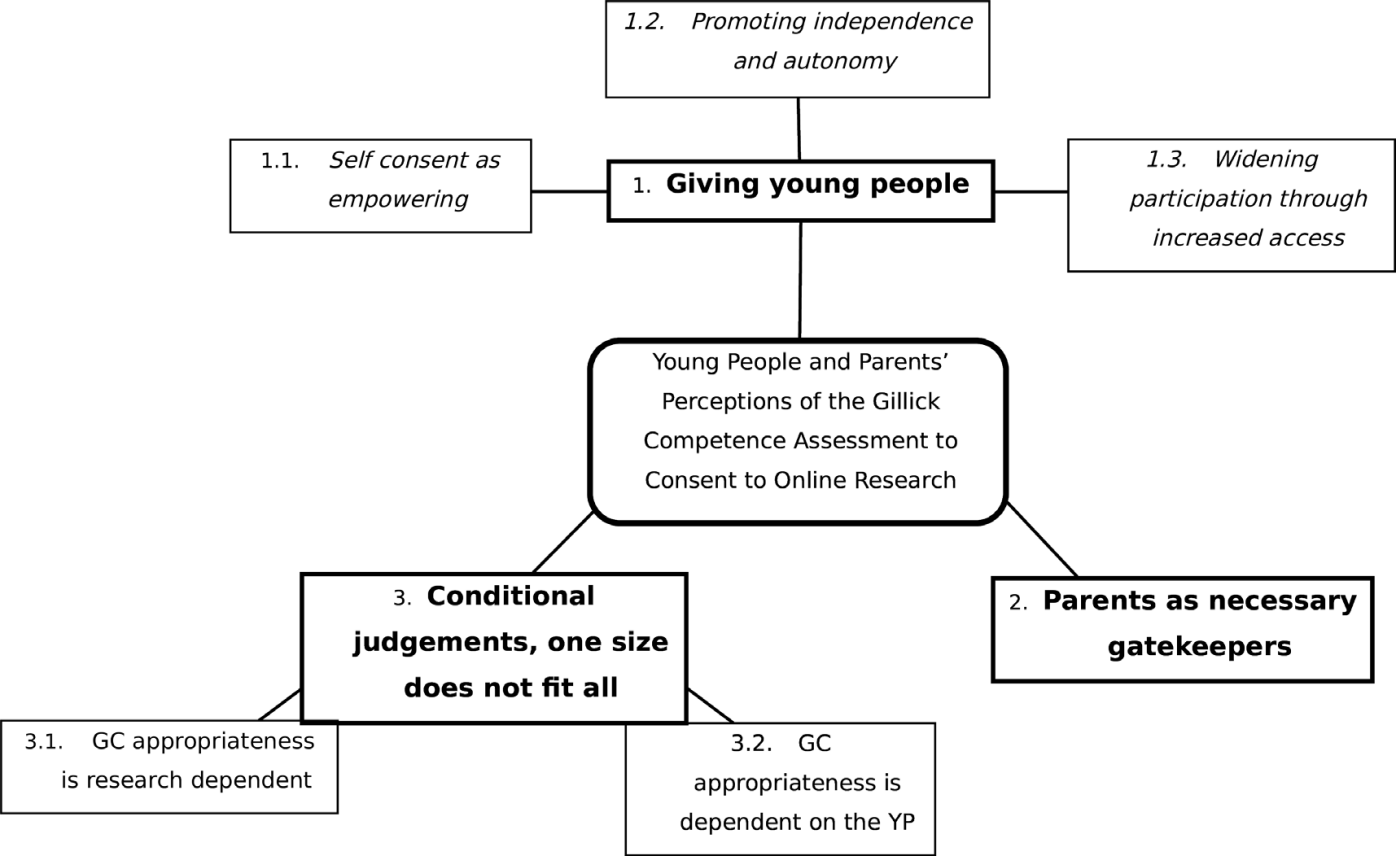
Thematic map. GC, Gillick Competence; YP, young people.

Reflexive thematic analysis of the data generated three themes which collectively explore YP’s and parental perspectives and attitudes towards the use of MCQs to allow 13–15-year-olds to demonstrate GC for consenting to participate in online research (see figure 1 for a thematic map and online supplemental table S4 for additional illustrative quotes).

### Theme 1: Giving YP a voice

YP and parents recognised the multiple benefits that the GC framework could afford YP, particularly in relation to empowerment, independence and widening access.

#### Self-consent as empowering

Participants described how the GC framework can be empowering as it provides YP with an opportunity to test their understanding of a study and determine whether they can consent for themselves. Additionally, participants shared how reading the information sheet and subsequently answering the MCQs could develop YP’s understanding of the study.

It’s meaningful because it’s helping check if you really understood what you went through. (Mia)

If you're aware of what it’s about and the content before, [YP are] more empowered to say yes or no. (Parent: Yasmin)

While most YP thought that the framework appropriately tested their understanding, some expressed concerns, emphasising that not all answer options were equally dismissible. Further, some felt like the MCQs were a memory test or *“a science test”* (Zach), which could trigger feelings of stress or confusion:

It’s quite scary doing multiple choice’ cause I was scared I was gonna get them wrong. (Max)

Some YP reflected that they felt “*under pressure*” (Anna) when completing the MCQs, resulting in a sensation of being “*in the spotlight*” (Max), although some suggested this could have been because they completed the questions on a video call. However, other YP felt “*relaxed*” (George) and “*calm*” (Peter) when answering the questions, indicating the diversity of experiences.

#### Promoting independence and autonomy

Participants reflected on how the GC framework may provide a bridge between childhood and adulthood, promoting independence and autonomy with a safety net. YP described the freedom afforded by being able to self-consent and contribute to something they are interested in or view as important, like mental health research.

It … gives it a feeling of independence…you’ve contributed to something that might cause change. Especially in mental health. (Anna)

YP also saw the framework as a way to take more responsibility and practise skills needed for adulthood. Parents agreed with this sentiment, believing the GC framework could enhance YP’s self-awareness and was reflective of elements of adult life, such as decision-making and cost-benefit analyses. Parents felt that these were fundamental lessons for YP to learn, and the framework could help to promote this.

It will make sure you can read something, interpret the information, and then … do something with that information, which is a skill that you need in life … to be a mature adult. (Jonah)

Parents also liked that the framework allowed YP to show whether they could consent for themselves, without completely ruling out their engagement if they cannot demonstrate GC. Parents liked this bespoke approach to consent rather than “*flicking the switch at 16*” (Parent: Rob).

#### Widening participation through increased access

Participants reflected on how, in some instances, requiring parental consent can be a barrier to research participation. Numerous potential reasons were stated, including strained relationships, parenting styles, time constraints and conflicting opinions.

I feel like some parents are more anxious, possibly more controlling, so you may then you know, get some parents who kind of would say no. (Parent: Olivia)

Both YP and parents indicated that certain topics may be more difficult to discuss with parents, including identity, mental health and relationships. Therefore, they believed that requiring parental consent might restrict and bias who takes part in research, as well as what YP might share within the research process.

Sometimes you find it … hard to speak to your parents about certain things, especially if you do not have this open relationship with your parent. (Mia)

Both YP and parents recognised the value that the GC framework could provide by increasing accessibility and reducing barriers for YP to consent, leading to more voices being heard and richer knowledge being gained.

### Theme 2: Parents as necessary gatekeepers

While participants recognised the benefits associated with the GC framework and the opportunity to self-consent, they also felt that parents played an important role in safeguarding YP against risks.

YP perceived parents as having more experience, and subsequently a better understanding and more awareness of the potential risks online and of taking part in research. As such, some YP indicated that they would still want to speak to their parents about the research, and they suggested that they should always be given an option to do so.

The benefit of like a parent getting involved always like kind of ensures safety of these kind of things. (Grace)

Parental perspectives on this matter differed depending on their perceived role as parents and the relationship they had with their child. Some parents felt that their role was about protection, having oversight and guiding their young person to make decisions that they are unable to make on their own. Parents who held these beliefs felt less confident in allowing the young person to consent for themselves.

For under sixteens, I think generally [parental consent is] a good thing mainly. Mainly because I think maybe it gives an opportunity for the parent and carer to confer with the child or young person over the purpose and the content of the study. (Parent: James)

Other parents felt as though their role was more to sit alongside the young person. They felt confident that they had raised an individual who could make decisions for themselves and wanted to encourage independence and transition away from requiring parental oversight. These parents felt the decision was dependent on how you raised your children, and whether they were raised to make sensible decisions.

The parents raise the children. The way they are dealing with each other, the way they are trusting each other, that’s very important … they will know their own children, and [whether] they have the right capacity to make the right choice for themselves. (Parent: Jane)

### Theme 3: Conditional judgements—one size does not fit all

Both parents and YP were hesitant to agree that the GC approach to self-consent would be appropriate in every situation for every individual. They highlighted the importance of considering the context of the research and of the young person hoping to participate.

#### GC appropriateness is research-dependent

Both parents and YP highlighted that the need for parental support in the consent process depended on the type of research being conducted and by whom. Parents seemed to feel reassured if research was conducted or endorsed by a reputable organisation of trained professionals, such as the National Health Service, or an educational establishment. This was partly because parents believed that large organisations would have well-established ethics committees in place to review and approve projects, meaning that decisions around safeguarding had been considered. YP echoed this trust in well-known organisations.

If it’s like from like some massive like company or like university … then it’s gonna be fine. (Zack)

The fact that it came from school, I would make some assumptions that school had done its due diligence. (Parent: Helen)

The topic being researched was another important consideration for the appropriateness of the GC framework. Specific topics that parents felt comfortable with were really varied and somewhat contradictory. While some parents felt comfortable with mental health research, others would not want their child to engage in research regarding mental health and/or self-harm without their oversight. There did not appear to be a consensus among parents about areas of research that were and were not acceptable.

Maybe research around self-harm makes, makes me feel anxious because I think there’s been a lot in the press hasn’t there about young people encouraging other young people to self-harm? (Parent: Olivia)

#### GC appropriateness is dependent on the YP

Across both participant groups, individual factors unique to each young person were considered highly influential in determining the appropriateness of the GC framework. YP and parents recognised that the 13–15-year-old age span is a crucial and rapid period of development where YP mature at different rates. Subsequently, while the GC assessment process might be appropriate for YP who have the cognitive capacity, emotional maturity and experience to weigh up decisions and think about the consequences, this might not universally be the case.

Some of the 13-year-olds, they quite mature cause it’s everybody’s developing different. I mean you know they develop different times, some of them mature some of them - even though they're 15-16 - they still feel very not mature not you know making the right choice for themselves. (Parent: Jane)

Within this, parents noted that it was extremely important to ensure that the reasons for YP not passing the assessment were not based on deficits related to memory or comprehension and/or language skills.

It massively makes me think about literacy skills and your diversity and how accessible that you know how. Yeah, just checking that the lack of understanding potentially isn't due to just not having been able to engage in like the kind of the language. (Parent: Cleo)

Some of these concerns were exemplified by the differing experiences of the YP completing the GC MCQs. Overall, participants found the questions well-structured and easy to understand, although views on the difficulty level of questions varied; for example, Daniel found that “*some of them seem a bit too easy*”, whereas Grace indicated that “*‘I struggled with it*”. Anna was surprised by how difficult answering the questions actually was in practice, in comparison to her prior expectation: “*‘it was a lot harder than I thought it was gonna be … I didn't actually take in as much as I thought*” (please see online supplemental material S4 for additional illustrative quotes).

## Discussion

Our exploratory study found that using MCQs to assess GC to enable 13–15-year-olds to self-consent to participate in an online, anonymous mental health intervention research study was acceptable to both YP themselves and to parents of this age group. YP wanted to have the option of trying to consent for themselves, and most participants demonstrated sufficient understanding of the study’s purpose, process, risks and benefits by answering the MCQs correctly. Both YP and parents perceived the GC assessment process as empowering but cautioned that its use must be within a robust framework of safeguards and highlighted the need for flexibility and accountability in research consent processes.

It is encouraging that the GC assessment process was perceived as empowering by both YP and parents. Importantly, it seemed to facilitate a deeper understanding as engaging with the MCQs helped YP better comprehend the study materials, potentially promoting more informed choices than widely used parental consent processes do. It is crucial to empower all YP to access mental health intervention research studies to ensure that research is as representative as possible of potential end users; giving YP the option of consenting to take part independently could overcome the barrier that requiring parental permission can pose for some YP.^21 37 38^ Potentially those who are also most vulnerable to developing mental health problems are also those who might be least likely to seek parental permission.^16 19 39^ By removing such barriers and enabling autonomous participation, while still ensuring that we do not just presume competence by waiving the requirement for parental consent, we can ultimately enhance the evidence base for early intervention efforts for mental health and better mental health outcomes.

Furthermore, responses given to the MCQs seemed to match narratively described understanding of the YP interviewed. Consistent with prior work,^5 9 10^ we found that some but not all YP aged <16 responded correctly. This may be because of individual differences in cognitive development and therefore in the ability to demonstrate understanding.^40^ Variability in MCQ response accuracy could also be due to the research procedure. In the current study specifically, which by necessity included an information sheet for the current study in addition to the information sheet for Project Care UK, to which the GC questions relate (due to the need to follow standard consent procedures), leading to potential confusion about which study information sheet we were referring to. This is likely to mean that our findings were conservative, and more YP could demonstrate GC when the GC assessment process is used as intended (ie, MCQs directly after the information sheet and pre-consent, with two attempts given). Another potential explanation for incorrect responses could be due to a lack of concentration or skipping through study materials. Therefore, it is important that YP who do not answer all four MCQs correctly on their first attempt get offered an opportunity to revisit the information sheet and given a second attempt at the MCQs, although offering more retake opportunities does increase the chances of getting it correct through guessing. It is also important that information sheets for studies are provided in a range of formats, including audio and/or audio-visual formats to enable YP to engage with them. This kind of material could be co-created with YP to maximise its relevance and appropriateness.

Parental perspectives in the current study highlighted the complexity of implementing a newly developed GC assessment process for online, anonymous studies. In the current study, the example study we used was Project Care UK, a self-help single-session intervention that is low risk, acceptable to YP and potentially effective at reducing mental health symptoms.^41^,^43^ Our intention is to make engaging, evidence-based help available on demand, for free, anonymously and on YP’s own terms. While parents supported the use of the GC assessment process in such studies, they were understandably more cautious about how this might be applied to higher risk and more complex studies. While parents expressed support for the idea, contingent on appropriate safety measures, perspectives were nuanced as views would depend on the nature of the research itself and who was conducting it, as well as what their intentions were. This is in line with previous research in the context of sexual minority youth^25^ and sexual health research.^44^ It is also consistent with wider discourse on issues related to children’s consent to medical procedures as well as to research studies.^3^

Although we specifically focused herein on consent to low-risk, mental health intervention research, it is important to also note that there is evidence that requiring parental consent to access mental health treatment has an impact on help-seeking. For instance, in the USA, in states where YP could not independently consent to mental health treatment, only 37% of those who experienced a depressive episode in the past year received any form of treatment, and while in states which allowed YP to consent for themselves with no or minimal restrictions, the treatment rate was higher, at 46%.^45^

### Strengths and limitations

Using a concrete example of an information sheet that was in use in an actual study (Project Care UK), from which we recruited some of our YP participants, is a strength of our method and improves the validity of our study. However, like many online studies advertising voucher incentives for study participants,^2946^,^48^ our Qualtrics project for sign-ups to the current qualitative study was affected by potential fraudulent responses, which meant that we had to introduce extra questions to answer, such as RECAPTCHA questions, which could have been off-putting. It also may be that in our subsequent manual checking process, we excluded some genuine YP who discontinued due to the MCQs being off-putting by erroneously concluding they were bots, although we think this is unlikely because of our systematic process for concluding that a potential participant was a likely bot/fraudulent responder, which included communicating by email with those whom we were uncertain about. The nature of our qualitative study also meant that the interview schedule was potentially confusing for YP as the GC MCQs which we asked about in the current study were about the Project Care UK study, but we had also provided them with information about the current study. We also addressed this by changing the order of the interview schedule in later interviews to improve clarity. Participants also provided feedback on how to further improve GC MCQs, which can be found in table 4.

**Table 4:** Participants’ suggested improvements to the Gillick Competence multiple-choice questions

Recommendations	Young people	Parents
*Information sheet*		
Less information so it is easier to read	X	X
Simpler language	X	X
Make the design more interesting and engaging	X	X
Make sure participants can go back and look at the information sheet if they get stuck	X	
Changing words like ‘intervention’ and ‘therapist’ as some young people may not know what this means—or adding clear definitions in bold or italics		X
*Question feedback*		
Make sure the response options are similar lengths	X	
You could consider using open questions to better assess understanding	X	
Make the design more interesting and engaging	X	X

## Conclusion

Our study provides preliminary evidence supporting the use of MCQs to assess GC in the context of low-risk, anonymous, online mental health intervention research for YP aged <16 in the UK. By enabling YP to demonstrate their understanding and consent independently, this approach has the potential to expand the reach of research studies, the representativeness of the evidence base and the effectiveness of resultant interventions. However, further research is warranted to explore the feasibility and validity of this approach for GC assessment to consent to participate in research studies, including across diverse populations and types of research studies, while also ensuring that appropriate safeguards are in place to protect the rights and well-being of YP.

## supplementary material

10.1136/bmjopen-2024-090747 online supplemental file 1

## Data Availability

Data are available upon reasonable request.
