# Supplementary material for: Consenting for themselves: a qualitative study exploring a Gillick Competence assessment to enable adolescents to self-consent to low-risk online research
Source: BMJ Open. 2025 Mar 4;15(3):e090747. doi: 10.1136/bmjopen-2024-090747 (PMC11883880; doi:10.1136/bmjopen-2024-090747)
Supplement: online supplemental file 1 [file bmjopen-15-3-s001.docx]

**Online Supplementary materials**

**S1. Original Young Person Interview Schedule**

Hello/Good Morning/Good Afternoon/Good Evening.

My name is xxxx, and I am a researcher at the University of Bath. Thank you for your time.

As you read in the information sheet when you signed up to the study, this interview will be recorded for research purposes and I will now switch on the recording and transcription. Remember, you can choose to stop taking part at any point, and you do not have to answer any questions that you might find uncomfortable. Anything that you say to me will be confidential, unless I have reason to believe that you may be a risk to yourself or others. All your answers will be anonymized, and you will not be able to be identified personally in any of our reports we write about what we find. Are you happy to continue and share your experiences for the purpose of this study?

The interview today will last between 20 and 40 minutes. It will be made up of three parts. For the first part, I will show you an information sheet about Project CARE-UK and then will ask you some multiple-choice questions about this. I will then ask you some more questions about your experience of answering the questions.

Okay, are you ready to get started?

Now I am going to share the information sheet about Project CARE-UK, and then I am going to ask you some multiple-choice questions about the information sheet.

Thank you.

I will now ask you some questions about your overall experience of reading that information and answering those questions. I will ask you about your understanding of Project CARE-UK. Then, I will share the multiple-choice questions again on my screen, one at a time, and ask you to tell and ask you to think aloud on your thoughts, feelings, and experiences of the question.

**Section 1: Overall Experience of the Gillick Competence Process**

In most studies of under 16-year-olds in the UK, parents are asked to give their consent (i.e. permission) for the young person to take part.

1. What do you think about whether parents should be asked?

Prompts:

- What do you think about whether young people themselves should be able to choose what studies they take part in and consent for themselves?
- *Certain topics may be more or less likely to ask your parents for consent for?*
- What problems are there with young people needing to ask their parents to consent?
- What are the problems with young people consenting for themselves?
- Potential benefits of young people consenting for themselves?

1. We asked you the multiple-choice questions to check that you understood what was in the information sheet. If young people can understand the information about a study, including what the study involves, why it is being done, and what the risks and benefits could be of taking part, then they can consent for themselves.

What did you think about having the multiple-choice questions to check for consent?

Prompts:

- Were there any things you would like to change?
- Any other benefits to the process?

**Section 2: Understanding of what Project-CARE UK involves**

1. From what you have read in the information sheet, why are we doing Project Care-UK?

- Prompt: What will we learn from it?

1. From what you have read in the information sheet, what does taking part in Project Care-UK involve?

- Prompt: What will you be asked to do if you take part?

1. From what you have read in the information sheet, what could the benefits be of taking part?

- Prompt: How will taking part help you and others?

1. From what you have read in the information sheet, what are the risks of taking part?

- Prompt: What are the bad things that could happen if you take part?

**Section 3: Think-Aloud Interview**

Main Prompt: I will now show you each of the 4 multiple choice questions you answered, one at a time. For each, I’d like you to tell me what you think of the question and of the response options.

Additional Prompts:

What were your thoughts and feelings while completing this question?

Feelings- anxious, confident, calm, any other emotions?

What did you think about this question?

What suggestions do you have to improve this question or the response options?

We are now nearing the end of this interview. Thank you for your participation. Is there anything else you’d like to share with me? Are there any other thoughts or concerns etc.

I will stop the recording now. I will email you some more details about this study and resources that you can contact in case you are experiencing any distress following this interview. In case you have any questions or would like more information on this study, feel free to get in touch with me through the contact details provided on the document.

As a thank you, we will send you a £10 amazon voucher. What email address would you like this to be sent to? Please remember to either send a screenshot confirming you have received the voucher or send us an email to confirm you have received it.

Thanks for taking part, and have a nice day!

**S2. Updated Young Person Interview Schedule**

Hello/Good Morning/Good Afternoon/Good Evening.

My name is xxxx, and I am a researcher at the University of Bath. Thank you for your time.

We are interested in hearing your views about a method we are developing to allow young people age under 16 to agree to take part in research studies without requiring their parent permission.

Legally, here in the UK, if a young person can demonstrate that they understand the purpose of a study, what it involves, and the risks and benefits of taking part, then they can consent themselves instead of asking their parents for permission. However, if there is no way to test this in online interventions and mental health research- so young people under the age of 16 always have to ask their parents for consent.

So that is why we are developing the method and we are interested in hearing your views about this.

The interview today will last between 30 and 40 minutes.

As you read in the information sheet when you signed up to the study, this interview will be recorded for research purposes and I will now switch on the recording and transcription. Remember, you can choose to stop taking part at any point, and you do not have to answer any questions that you might find uncomfortable. Anything that you say to me will be confidential, unless I have reason to believe that you may be a risk to yourself or others. All your answers will be anonymized, and you will not be able to be identified personally in any of our reports we write about what we find. Are you happy to continue and share your experiences for the purpose of this study?

Okay, are you ready to get started?

There are three sections to today. For the first, I will ask you some questions on what you think about whether young people aged 13-15 should be able to consent to research studies for themselves without asking their parents.

**Section 1: Overall Experience of the Gillick Competence Process**

In most studies of under-16-year-olds in the UK, parents are asked to give their consent (i.e. permission) for the young person to take part.

What do you think about whether parents should be asked?

Prompts:

- What do you think about whether young people themselves should be able to choose what studies they take part in and consent for themselves?
- *Certain topics may be more or less likely to ask your parents for consent for?*
- What problems are there with young people needing to ask their parents to consent?
- What are the problems with young people consenting for themselves?
- Potential benefits of young people consenting for themselves?

**Section 2**

Today, we are going to show you an information sheet containing information of a different study we are doing in Project Care- UK. We are using this to test out our method.

**Present Information sheet**

**Present Multiple-Choice Questions**

**Understanding of what Project-CARE UK involves**

- From what you have read in the information sheet, why are we doing Project Care-UK?
  - Prompt: What will we learn from it?
- From what you have read in the information sheet, what does taking part in Project Care-UK involve?
  - Prompt: What will you be asked to do if you take part?
- From what you have read in the information sheet, what could the benefits be of taking part?
  - Prompt: How will taking part help you and others?
- From what you have read in the information sheet, what are the risks of taking part?
  - Prompt: What are the bad things that could happen if you take part?

I will now ask you some questions about your overall experience of reading that information and answering those questions.

We asked you the multiple-choice questions to check that you understood what was in the information sheet. If young people can understand the information about a study, including what the study involves, why it is being done, and what the risks and benefits could be of taking part, then they can consent for themselves.

What did you think about having the multiple-choice questions to check for consent?

Prompts:

- Experience of reading the information sheet?
- Answering the questions?
- Were there any things you would like to change?
- Any other benefits to the process?

**Section 3: Think-Aloud Interview**

We are now going to do a think- aloud interview where we will ask you to verbalise your thoughts and feelings to each of the multiple choice questions- just want to hear what is going through your mind as you look at the questions.

Main Prompt: I will now show you each of the 4 multiple choice questions you answered, one at a time. For each, I’d like you to tell me what you think of the question and of the response options.

Additional Prompts:

- What were your thoughts and feelings while completing this question?
- Feelings- anxious, confident, calm, any other emotions?
- What did you think about this question?
- What did you think about the difficulty level?
- Language? Content? How it looks?
- What are your thoughts on the options/ the difference between them?
- What suggestions do you have to improve this question or the response options?

We are now nearing the end of this interview. Thank you for your participation. Is there anything else you’d like to share with me? Are there any other thoughts or concerns etc.

I will stop the recording now. I will email you some more details about this study and resources that you can contact in case you are experiencing any distress following this interview. In case you have any questions or would like more information on this study, feel free to get in touch with me through the contact details provided on the document.

As a thank you, we will send you a £10 amazon voucher. What email address would you like this to be sent to? Please remember to either send a screenshot confirming you have received the voucher or send us an email to confirm you have received it.

Thanks for taking part, and have a nice day!

**S3. Parent Interview Schedule**

Hello /Good Morning/Good Afternoon/Good Evening.

My name is xxxx, and I am a researcher at the University of Bath.

Thank you again for agreeing to take part. We are interested in hearing your views about a method we are developing to allow young people age under 16 to agree to take part in research studies without requiring their parent/caregiver’s permission. Legally, here in the UK, if a young person can demonstrate that they understand the purpose of a study, what it involves, and the risks and benefits of taking part, then they can consent themselves as they are deemed ‘Gillick competent’. In practice, most studies, and especially online survey studies, ask young people to ask their parents to tick the box to agree for them to take part rather than assessing Gillick competence and proving legitimacy of this is difficult and the models can pose ethical challenges (Brothers et al., 2020).

As part of developing this process, it is important that we understand how parents feel about this and what they think. So, I will begin by asking you some general questions about your thoughts about young people agreeing to take part in studies for themselves, and Gillick competence. Then I will show you the information sheet for a specific project we are doing, and the multiple-choice questions we have developed to assess a young person’s Gillick competence. For each of these, as I screen share it with you, I will ask you to tell me your thoughts, feelings, and experiences of the question.

Just to remind you, this interview will be recorded for research purposes, and I will now switch on the recording. Remember, you can choose to stop taking part at any point, and you do not have to answer any questions that you might find uncomfortable. Anything that you say to me will be confidential, unless I have reason to believe that you may be a risk to yourself or others. All your answers will be anonymized, and you will not be able to be identified personally in any of our reports we write about what we find. Do you consent to continue and share your experiences for the purpose of this study?

**Section 1: Overall Experience of the Gillick Competence Process**

In most studies of under 16-year-olds in the UK, parents are asked to give their consent (i.e. permission) for the young person to take part.

1. What do you think about whether parents should be asked?

Prompts: What do you think about whether young people themselves should be able to choose what studies they take part in and consent for themselves? What problems are there with young people needing to ask their parents to consent? What are the problems with young people consenting for themselves?

1. What we are planning to do in our study is to give young people information about the study in an information sheet, and then to check if a young person understands the information, including what the study involves, why it is being done, and what the risks and benefits could be of taking part. We will do this by asking multiple choice questions. If they get them all right, then they can consent for themselves. If they get one or more wrong, they will be prompted to read the information sheet again, and given a second chance to answer the questions. If they get one or more wrong again, they will not be able to proceed to consent for themselves but could ask for parental permission instead. What do you think of this idea?

Prompts: What are your concerns about us doing this?

What do you feel the benefits to this process are?

What aspects do you think are important to consider when developing this process?

**Section 2: Understanding of what Project-CARE UK involves.**

Please glance through this information sheet (SHARE IN CHAT).

**Section 3: Think-Aloud Interview**

Main Prompt: I will now show you each of the 4 multiple choice questions we have developed for young people to answer. I will show these one at a time. For each, I’d like you to tell me what you think of the question and of the response options.

Additional Prompts:

What were your thoughts and feelings about this question?

What did you think about this question?

What suggestions do you have to improve this question or the response options?

We are now nearing the end of this interview. Thank you for your participation. Is there anything else you’d like to share with me?

I will stop the recording now. I will email you a document with some more details about this study and resources that you can contact in case you are experiencing any distress following this interview.

In case you have any questions or would like more information on this study, feel free to get in touch with me through the contact details provided on the document.

Table S4. Additional illustrative quotes by theme

| **Theme** | **Subtheme** | **Quotes** |
| --- | --- | --- |
| Theme 1: Giving YP a voice | 1.1 Self-consent as empowering | *“I think it was definitely like good in the sense of testing someone’s understanding.”* (Grace, 15)  *“That is a good way to see how people kind of process what they’re reading because well then you find out what they’ve taken in.”* (Anna, 15)  *“As you go through it…you understand the benefit of it and what is required.”* (George, 14)  *“It will make sure [young people] know what they’re getting themselves into.”* (Lily, 13)  *“A benefit I feel from being involved in research and studies, is there's a huge amount of learning even just going through those processes, being involved in talking to people. If those are safe environments and things.”* (Parent: Anne, 13)  *“I actually do feel strongly that young people should be able to make their own decisions and be autonomous in that.”* (Parent: Anne, 13) |
|  | 1.2 Promoting independence and autonomy | *“By allowing teenagers to start consenting to things, it allows them to develop…sort of merge into becoming an adult.”* (Peter, 14)  *“It can help them when they do become an adult.”* (Daniel, 14)  *“It could like make them more independent…and give them a bit more freedom.”* (Lily, 13)  *“I think you know, trying to credit young people with, with more independence and more more of a say.”* (Parent: Rob, 14)  *“Yeah, there are many benefits, one is that they are able to understand the surrounding and how to deal with all that stuff alone where the parents are not around to say. Second, it brings about self-awareness and how to handle it and how to handle the situation.”* (Parent: Matthew, 14) |
|  | 1.3 Widening participation through increased access | *“Maybe they’ll have the chance to say what they want to.”* (Rachel, 14)  *“Maybe if parents are a bit sceptical about the idea of being in research or their child being a part of research could stop it.”* (Anna, 15)  *“Maybe you got some key worker parents…they're always working and they don't really have the time, but…you can go and do it.”* (Jonah, 14)  *“It just means that more people are going to be able to access these things.”* (Jack, 15)  *“I think well, for one, it's really, really important to hear what young people have got to say; you might get less inhibited answers if a young person is doing it without their parents kind of overshadowing.”* (Parent: Nicola, 14)  *“I suppose, especially because at 13, 14, 15 they're gaining a lot more independence. And kind of, you know, developing their self-identity and. I think that could be really it's it's a nice. Maybe it could be seen as a kind of a nice opportunity for them to take part in that and influence, influence research that is about them is about young people. I just think if I was, you know child with some issues about gender etcetera, I'd feel quite “supported, I'm involved”. It's quite empowering.”* (Parent: Sophie, 14) |
| Theme 2: Parents as necessary gatekeepers | | *“It can help help the child to know if it's safe to do.”* (Damien, 13)  *“They're able to gauge situations better…they've got more life experience so they can be like you know what…I don't think this is appropriate.”* (Grace, 15)  *“Parents are probably gonna be more mature to know what they’re doing.”* (Zack, 15)  *“I guess one of the things I'm thinking about is risk in terms of whether the the research if it if it's about something that could trigger something with the child. That potentially could then lead to something stressing for them. If as a parent, if you're not kind of aware that that's happening. It feels that feels tricky, you know, because usually as a parent or certainly my child is 13, so I feel they're still quite young, so you know you want to know everything that's going on with them. And so you can look after them.”* (Parent: Olivia, 13) |
| Theme 3: Conditional judgements: One size does not fit all | 3.1 GC appropriateness is research-dependent | *“If… I’ve really strongly feeling about something that you're putting research into, then maybe it could be OK because…you're more likely to fully try and see…what you're doing, but if…you don't really care I think…someone else should tell you what they think, because like, you won't really look at what you are doing.”* (Anna, 15)  *“If it's coming from NHS. It's a big organisation, you have trust in them. But if it's coming from some other agency that I didn't know, then I wouldn't, you know, maybe be a bit more wary, yeah.”* (Parent: Sophie, 13)  *“I think in terms of the kind of harms, I think it depends on if we have better structures and system. You know the ethics around it. If that's really robust then.”* (Parent: Anne, 14)  *“If it can be proved to be safe, and obviously you've got your safeguards, your end, at the academic end because you have to go through ethics committees. ... that’s fine.”* (Parent: James, 14)  *“For this, mental health, I think they can decide for themselves to take part, because for the mental health it’s like different age have different understanding – am I understanding this good – they can make consent for them to take, yeah. I think in this situation, don’t need the parents to consent because they can do it for themselves for this research.”* (Parent: Jane, 14) |
|  | 3.2 GC appropriateness is dependent on the YP | *“Some people are definitely more able to…make decisions for themselves.”* (Grace, 15)  *“It depends on the teenager, but according to me I say I can consent for myself, without depending on the parent. Then when I think about other teenagers, maybe someone needs some guidance at least, for them to understand the information clearly.”* (Tom, 15)  *“Some young people might need extra help from their parents or might not fully understand it.”* (Lily, 13)  *“It depends because some kids at age 12 they're much more matured than someone who's in their mid-teens who's just having a laugh or doing something silly.”* (Peter, 14)  *“They have such different life experiences and that will kind of impact the way that they're responding and also the way that they relate to the person doing the research.”* (Parent: Cleo, 15)  *“You find that if you come to ask a given child about a given situation, maybe you have gone through the trauma. And you want he or she won't be comfortable to talk about it or it will bring back memories which will hunt him or her down, it literally depends on the background.”* (Parent: Matthew, 14)  *“What it doesn't factor in is the emotional competence of a of a young person. And that is something that, you know, they might be, like I said before it they can read it, they can draw out the relevant information like a quiz almost, you know.”* (Parent: Nicola, 14) |

*Note: GC = Gillick Competence; YP = Young people.*
