# Supplementary material for: Consenting for themselves: a qualitative study exploring a Gillick Competence assessment to enable adolescents to self-consent to low-risk online research
Source: BMJ Open. 2025 Mar 4;15(3):e090747. doi: 10.1136/bmjopen-2024-090747 (PMC11883880; doi:10.1136/bmjopen-2024-090747)
Supplement: Supplementary file 2 [file bmjopen-2024-090747.reviewer_comments.pdf]

PEER REVIEW HISTORY

BMJ Open publishes all reviews undertaken for accepted manuscripts. Reviewers are asked to complete a checklist review form and are provided with free text boxes to elaborate on their assessment. These free text comments are reproduced below.

ARTICLE DETAILS

Title (Provisional)

Consenting for themselves: A qualitative study exploring a Gillick Competence assessment to enable adolescents to self-consent to low-risk online research

Authors

Loades, Maria; Willis, Lara; Wilson, Emma; Perry, Grace; Luximon, Melanie; Chiu, Christy T.C.; Higson-Sweeney, Nina

---

VERSION 1 - REVIEW

**Reviewer**	**1**
**Name**	**Sutcliffe, Katy**
**Affiliation**	**University College London Institute of Education, Social Science**
**Date**	**12-Aug-2024**
**COI**	**I have no competing interests.**

A really interesting study with an innovative approach to facilitate YP to consent to and participate in research studies. This is important and useful work that could transform online research with children and help to challenge outdated notions about children's ability to consent. However, the complexity of the design and the topic mean that further efforts to enhance clarity are essential.

It is a multi-component mixed-method study which is also a study within a study i.e. a mixed methods study embedded within a single arm pre-post intervention program evaluation - Project Care UK. Only the qualitative aspect of the pilot study is referred to as a 'sub-study' but both the quantitative and qualitative elements of this pilot study could be considered 'sub-studies' of the main Project Care UK intervention evaluation. The elision of the quantitative aspects of this pilot study with the main intervention evaluation mean the communication of methods for quantitative aspects of the pilot are unclear at points.

Additionally, the study is focused on consent to research and there is occasionally confusion between consent to research and consent to medical treatment.

These complicated elements of the research design and topic mean that clear communication is absolutely vital. Below I have indicated some places where revisions are needed. I strongly encourage the authors to revise and resubmit.

Title:

Consenting for themselves: A mixed methods pilot study exploring a Gillick Competence process to enable adolescents to self-consent to online research - It would be helpful for readers if the title makes clear that young people are consenting to 'low-risk' online research. Also suggest to change 'gillick competence process' to 'gillick competence assessment'.

Abstract:

Page 1 – lines 15-22: The background section of the abstract is unclear whether your interest is in consent to research or consent to a treatment/ intervention - from reading the paper I think it's about consent to research not consent to digital mental health interventions. The first couple of sentences suggest you are talking about parental consent being a barrier to receiving digital mental health interventions – rather than a barrier to participation in research: 'Providing digital mental health interventions online could expand access to help for young people. However, requiring parental consent may be a barrier to participation.' The later sentence then talks about consent to research 'self-consent to online, anonymous, low-risk studies.' Suggest to make clearer that your interest is in consent to research.

Page 1 – line 23: 'Aims: We aimed to pilot such a process to establish whether it is feasible and acceptable to both young people and parents.' The research aims should be a stand-alone statement so suggest to remove the phrase 'such a process' and be more explicit about what process you are referring to – e.g. 'We aimed to pilot a method for assessing Gillick Competence to participate in online studies to establish whether it is feasible and acceptable to both young people and parents.'

Page 1 – lines 27-30: 'Using a mixed methods approach, we developed a series of multiple-choice questions (MCQs) to assess understanding of a specific online self-help study testing a self-kindness intervention (Project Care UK).' The sentence suggests that a mixed-method approach was used to *develop* the MCQ –from having read the paper I think the mixed-method approach was only used to *evaluate* the feasibility and acceptability of the MCQ? Suggest to revise to something like: 'Using a mixed methods approach we evaluated the acceptability and feasibility of a multiple-choice question tool designed to assess understanding of a specific online self-help study …'. Also, since you have said 'using a mixed methods approach' the sentence 'we collected qualitative and quantitative data' could be dropped?

Page 1 – lines 27-35: I realise here and in the main body of the report that I'm not clear what the 'quantitative data' comprises. In the abstract you refer only to quantitative data in terms of answers to the MCQs. But in the main body of the report you also mention that some quantitative data is collected from a series of questions asked directly after consent to assess interest in the opportunity to show GC, interest in using online tool to show GC (lines 51-60 on page 4). This second type of quantitative data is not mentioned in the abstract. If quantitative data comes both from answers to MCQ and questions about acceptability of the MCQ I would suggest to make this clearer.

Introduction:

There are a couple of points in this section that would benefit from further clarification to do the concept of children's consent justice.

Page 3 lines 12-15: You suggest that 'competence *must* be proven or consent sought from an individual with caring or parental responsibilities' (see comments on refining this sentence in minor points section). It would be worth indicating who has determined that this 'must' happen – are you referring to the declaration of Helsinki? The adoption of Helsinki by international organisations such as the World Medical Association? The legal framework(s) in specific countries?

Page 3 Line 12: 'competent of making that decision' should read 'competent to make that decision'

Page 3 lines 12-15 – the following sentence doesn't quite make sense: 'Competence to consent in research tends to be assumed in adults, but in minors (i.e., anyone under the legal age of adulthood) must be proven or sought from an individual with parental or caring responsibilities (henceforth 'parents').' - i.e. 'competence must be proven or consent sought ...'

Page 3 lines 16-17: Children are not always asked to provide 'assent' and if you are to include the idea you would need to define what 'assent' means. The concept is contentious – see this paper by Priscilla Alderson - Children's consent and the zone of parental discretion - P Alderson, 2017 (sagepub.com). Alderson is a key writer in the field of children's consent and it would be worth engaging with more of her work in the introduction section.

Page 3 line 28: I suggest a couple of amendments for this sentence: 'GC means that YP can evidence that they understand the purpose of the study, what taking part will involve, and the potential risks and benefits of participation'.

As it stands this sentence implies that GC is specific to research. It is only later that you explain that GC actually relates to consent to medical treatment. If readers miss the later context they may assume that GC relates only to research. I suggest being clear in this sentence that it refers to consent to medical treatment – and I strongly suggest to use the exact wording of Lord Scarman about what it actually means – i.e. : whether a child has 'a sufficient understanding and intelligence to enable him or her to understand fully what is proposed' and 'has sufficient discretion to enable him or her to make a wise choice in his or her own best interests'.

And then you could make clearer that you have interpreted GC in relation to research – e.g. continue with something like the following: '*In the context of research GC may therefore be interpreted as meaning* that YP can evidence that they understand the purpose of the study …'

Page 3 – lines 32-37: 'Available evidence indicates that the ability to understand medical decisions among YP who are ≥13 years old is similar to that of adults (5), although there is considerable individual variability in cognitive, socio-emotional and neurological development (6-8). Therefore, GC cannot simply be presumed.' A couple of suggestions re this sentence.

First, the final sentence 'GC cannot simply be presumed' doesn't quite reflect the line of argument in the preceding sentences – the evidence you give suggests that you're not just arguing that GC cannot be presumed, rather that evidence suggests many children and young people may have the competence to participate, and therefore we need means of assessing that?

Also, in case of interest, this paper provides evidence that even younger children are presumed competent to participate in medical decisions - Children's ages of consent to non-urgent heart surgery: The views of two paediatric cardiology teams - Alderson - 2024 - Children & Society - Wiley Online Library

Page 3 – lines 44-45: 'These practices are not typically followed in online studies, which tend to opt to seek parental consent for <16's.' This statement needs a citation to verify it.

Page 3 line 54 - page 4 line 10: This section is very much focused on concepts such as reliability and validity – 'representation and generalizability but can also impact the validity and reliability of a study' but these concepts relate primarily to quantitative rather than qualitative research. You might also consider children's right to participate in research, and in general the important knowledge lost from the most marginalised groups – see e.g. this paper which argues for the 'undeniable need to

listen to children and adolescents as members of society; consider children's rights to actively participate, and voice their beliefs, opinions and experiences for methodological reasons, as it could enhance the quality and use of knowledge produced for the common good.' Children and adolescents' voices and the implications for ethical research - Hayda Alves, Lisa Gibbs, Katitza Marinkovic, Irma Brito, Payam Sheikhattari, 2022 (sagepub.com)

Page 4 - lines 12-20: 'Although the option of GC has yet to become common practice within online research' – This sentence could be clearer - GC referes to 'Gillick Competence' rather than assessment of GC – i.e. you could say something like 'the option of *assessing* GC has yet to become common practice within online research'? And later 'an approach has been developed in Australia that allows <16s to consent to online research participation through *assessing* GC using a multiple choice test before *signing* the consent form.'

Methods

Page 4 – line 44-45: 'to assess comprehension' – please could you add comprehension of what? –

e.g. 'to assess comprehension of the study information and the implications of participation'?

Page 6 – lines 9-10: I was not clear how people were recruited to the quantitative part of the study – I think this sentence may be describing that but could be clearer - 'routed them to a separate Qualtrics survey from Project Care UK' – does the 'separate study' refer to this study? i.e. people were routed to answer the MCQs and answer questions about their interest in showing GC / using an online tool to show GC? I think my confusion relates to the fact that you only frame the qualitative work as a 'sub-study' and not the quantitative aspects of the pilot? Might it be better to frame the pilot as a 'mixed-methods sub-study' within the Project Care UK intervention evaluation?

Page 6 – line 19: 'After Qualtrics responses for bots' – I don't understand what this sentence means? Are there some missing words here? What does 'for bots' mean? e.g. (assuming I've understood correctly) you could say something like - 'After checking Qualitrics using RECAPTCHA to filter out any responses by bots (automated rather than human survey responses) …'?

Page 6 – line 20: 'Semi-structured interviews incorporating think-aloud techniques' –a citation is needed here for 'think-aloud techniques' – you provide one later but good to include it here too.

Results

The section on the quantitative results is quite brief – but I can't really see what could be added. The reporting of the qualitative findings is nicely detailed. My one key comment would be to include a bit more commentary on the recommendations for improvement (currently only reported in table 5).

This could also be picked up on in the discussion.

Discussion

This work is clearly badged as a pilot study and conclusions are appropriately cautious – but a couple of areas of the discussion could be clearer and a little more consideration of limitations would be welcome.

Page 15 – lines 42-45: This section is really not clear what the 'qualitative sub-study' refers to – I had assumed you meant just the qualitative interviews – but this line 'However, both the main Project Care UK study and the qualitative sub-study were affected by fraudulent responses, and we used both automated and manual checks to discount likely bot responses (details on request).' i.e. I can't see how the qualitative interviews could have been affected by fraudulent responses / bots – so when you refer to the 'qualitative sub-study' are you including the survey / MCQs?

Page 15 – lines 51-52: 'but we had just provided them with information and consented them to the sub-study'. Sorry this is a real bug-bear of mine and a common problem but researchers can't 'consent participants' – participants provide consent to take part in research – so suggest to reword

e.g. 'But we had just provided them with information and they had consented to take part.'

Page 6 line 12: 'chance to register their interest to participant through' should read participate not participant.

Page 16 – lines 14-15: What is meant here by 'research settings' are you referring to more complex or higher risk research? If so perhaps make that clearer. And also what is meant by 'effectiveness' do you just mean acceptability and feasibility across diverse populations and research contexts? Or do you mean that testing 'effectiveness' is needed? If so effectiveness of what?

**Reviewer**	**2**
**Name**	**Fenton, Clare**
**Affiliation**	**Leeds and York Partnership NHS Foundation Trust**
**Date**	**23-Nov-2024**
**COI**	**None**

A much needed study in an under researched area, however, I do have some significant concerns and suggested revisions.

1: "The semi-structured interview was piloted with a young person (aged 16) to provide feedback, which resulted in changes to improve the clarity and flow of the interview schedule." It would be helpful to expand on what was changed and whether that was directly from feedback from the young person or from what was observed in the pilot interview by the researchers.

2: The MCQ development is a critical aspect of this study. It would be helpful to expand further on how they were developed initially from the previous research, including more information about the PPI conducted with young people (eg number of young people, ages, how many drafts they reviewed)

3: I note a convergent mixed method approach. It would be helpful to understand the rationale for a convergent approach.

4: There was a very large number of forms removed from analysis in data cleansing, some consideration about whether all of these were bots or some participants would be helpful. For example is it possible that some young people did not complete due to being unable to answer the MCQs?

5: It needs to be clearer whether the interviews were conducted with parents present or not and the rationale if it was with parents.

6: Only 18 young people under 16 completed the survey, the vast majority identified as white british which is a significant limitation and I have to question the results with such a limited sample size

7: Statistical analysis was conducted but there is no mention of this in the methodology

8: Further rationale needed around why the 16-18 year age group was included in the survey.

9: Changing the interview due to confusion noticed is appropriate but suggests that the interview was not sufficiently piloted before being deployed.

10: Objective 1 has not been met as no inference can be made about the proportion

11: Due to the concerns above I am not sure the conclusion is correct, it could also have been concluded that this was evidence that parental consent continues to be required and may be needed in those over 16 from the quantitative results.

12: The strength of this study is the qualitative work, maybe the study would be best reframed with emphasis on this and discussing how the interviews could inform a further review of how to consent young people under 16 (eg using a creative approaches such as videos to provide accessible information)

VERSION 1 - AUTHOR RESPONSE

Reviewer: 1

Thank you for your encouraging and useful overall comments, which we have not repeated here.

Title: Consenting for themselves: A mixed methods pilot study exploring a Gillick Competence process to enable adolescents to self-consent to online research - It would be helpful for readers if the title makes clear that young people are consenting to 'low-risk' online research. Also suggest to change 'gillick competence process' to 'gillick competence assessment'.

On reflection, we agree with these points and have revised our title as follows (pg.1):

Consenting for themselves: A qualitative study exploring a Gillick Competence assessment to enable adolescents to self-consent to low-risk online research

Abstract: Page 1 – lines 15-22: The background section of the abstract is unclear whether your interest is in consent to research or consent to a treatment/ intervention - from reading the paper I think it's about consent to research not consent to digital mental health interventions. The first couple of sentences suggest you are talking about parental consent being a barrier to receiving digital mental health interventions – rather than a barrier to participation in research: 'Providing digital mental health interventions online could expand access to help for young people. However, requiring parental consent may be a barrier to participation.' The later sentence then talks about consent to research 'self-consent to online, anonymous, low-risk studies.' Suggest to make clearer that your interest is in consent to research.

Thank you for drawing our attention to the need to clarify this distinction. Our abstract now reads as follows (pg.1):

Background: *Providing digital mental health interventions online could expand access to help for young people, but requiring parental consent may be a barrier to participation. We therefore need a method that enables young people <16 years old (i.e., presumed competent in the UK) to demonstrate Gillick Competence (understanding of purpose, process, potential benefits, and potential harms) to self-consent to online, anonymous, low-risk studies.*

Aim: *To explore whether a new method for assessing Gillick Competence to participate in low-risk, anonymous online studies is acceptable to both young people and parents.*

Method: *We interviewed 15 13–15-year-olds and 12 parents of this age group in the UK. Using a qualitative approach, we explored the acceptability of a series of multiple-choice questions (MCQs) designed to assess understanding of a specific online self-help research study testing a self-kindness intervention.*

Results: *The MCQ answers that participants gave mostly corresponded with their narrative explanations of their understanding during interviews. Young people and parents thought that the process was empowering and could increase access to research whilst also promoting independence. However, they emphasised the importance of individual differences and different research contexts and highlighted the need for safeguards to be in place.*

***Conclusions**: The MCQs were acceptable to both young people and parents, providing preliminary evidence for the potential of this process for allowing <16's to self-consent to online, anonymous, low-risk mental health research. Further research is needed to validate the effectiveness of this process among a diverse range of populations and research contexts.*

Page 1 – line 23: 'Aims: We aimed to pilot such a process to establish whether it is feasible and acceptable to both young people and parents.' The research aims should be a stand-alone statement so suggest to remove the phrase 'such a process' and be more explicit about what process you are referring to – e.g. 'We aimed to pilot a method for assessing Gillick Competence to participate in online studies to establish whether it is feasible and acceptable to both young people and parents.'

We have updated our aims in line with your comments as per the above revised abstract, and we agree that this will improve the clarity of this section.

Page 1 – lines 27-30: 'Using a mixed methods approach, we developed a series of multiple-choice questions (MCQs) to assess understanding of a specific online self-help study testing a self-kindness intervention (Project Care UK).' The sentence suggests that a mixed-method approach was used to develop the MCQ –from having read the paper I think the mixed-method approach was only used to evaluate the feasibility and acceptability of the MCQ? Suggest to revise to something like: 'Using a mixed methods approach we evaluated the acceptability and feasibility of a multiple-choice question tool designed to assess understanding of a specific online self-help study …'. Also, since you have said 'using a mixed methods approach' the sentence 'we collected qualitative and quantitative data' could be dropped?

This has been revised as follows, in line with your comments and the wider changes made in response to your and the 2nd reviewer's comments as per our revised abstract above.

Page 1 – lines 27-35: I realise here and in the main body of the report that I'm not clear what the 'quantitative data' comprises. In the abstract you refer only to quantitative data in terms of answers to the MCQs. But in the main body of the report you also mention that some quantitative data is collected from a series of questions asked directly after consent to assess interest in the opportunity to show GC, interest in using online tool to show GC (lines 51-60 on page 4). This second type of quantitative data is not mentioned in the abstract. If quantitative data comes both from answers to MCQ and questions about acceptability of the MCQ I would suggest to make this clearer.

Many thanks for highlighting the potential for confusion in the way we had described our mixed methods approach. This has been resolved now that we have removed the quantitative data from the manuscript, based on reviewer feedback.

Introduction:

Page 3 lines 12-15: You suggest that 'competence must be proven or consent sought from an individual with caring or parental responsibilities' (see comments on refining this sentence in minor points section). It would be worth indicating who has determined that this 'must' happen – are you referring to the declaration of Helsinki? The adoption of Helsinki by international organisations such as the World Medical Association? The legal framework(s) in specific countries?

We have reworded this as follows (pg.3):

The legal position, which does vary from country to country, tends to be that competence to consent to take part in research studies is assumed in adults, whereas in minors (i.e., anyone under the legal

age of adulthood), competence is not presumed (3). Therefore, consent is commonly sought from an individual with parental or caring responsibilities (henceforth 'parents').

Page 3 Line 12: 'competent of making that decision' should read 'competent to make that decision'

Thanks for noticing this, it has been changed accordingly.

Page 3 lines 12-15 – the following sentence doesn't quite make sense: 'Competence to consent in research tends to be assumed in adults, but in minors (i.e., anyone under the legal age of adulthood) must be proven or sought from an individual with parental or caring responsibilities (henceforth 'parents').' - i.e. 'competence must be proven or consent sought ...'

We have added these additional words to contextualise the sentence, it now reads (pg.3):

The legal position, which does vary from country to country, tends to be that competence to consent to take part in research studies is assumed in adults, whereas in minors (i.e., anyone under the legal age of adulthood), competence is not presumed (3). Therefore, consent is commonly sought from an individual with parental or caring responsibilities (henceforth 'parents').

Page 3 lines 16-17: Children are not always asked to provide 'assent' and if you are to include the idea you would need to define what 'assent' means. The concept is contentious – see this paper by Priscilla Alderson - Children's consent and the zone of parental discretion - P Alderson, 2017 (sagepub.com). Alderson is a key writer in the field of children's consent and it would be worth engaging with more of her work in the introduction section.

Thank you for signposting us to this interesting work. We have, on balance, decided to remove the mention of assent here as it does not seem pertinent to the argument we are making about competence to consent.

Page 3 line 28: I suggest a couple of amendments for this sentence: 'GC means that YP can evidence that they understand the purpose of the study, what taking part will involve, and the potential risks and benefits of participation'. As it stands this sentence implies that GC is specific to research. It is only later that you explain that GC actually relates to consent to medical treatment. If readers miss the later context they may assume that GC relates only to research. I suggest being clear in this sentence that it refers to consent to medical treatment – and I strongly suggest to use the exact wording of Lord Scarman about what it actually means – i.e. : whether a child has 'a sufficient understanding and intelligence to enable him or her to understand fully what is proposed' and 'has sufficient discretion to enable him or her to make a wise choice in his or her own best interests'. And then you could make clearer that you have interpreted GC in relation to research – e.g. continue with something like the following: 'In the context of research GC may therefore be interpreted as meaning that YP can evidence that they understand the purpose of the study …'

Thank you for your detailed comment. We agree that it is appropriate to make this section clearer. Based on your comments, we have added a sentence to clarify that GC was originally established in relation to medical treatment. We have also added the first quotation from Lord Scarman, as we believe that this provides a sufficient description of GC (pg.3):

GC was primarily established so that YP can consent to medical treatments. GC refers to whether a child has a "sufficient understanding and intelligence to enable them to understand fully what is proposed" (4).

Page 3 – lines 32-37: 'Available evidence indicates that the ability to understand medical decisions among YP who are ≥13 years old is similar to that of adults (5), although there is considerable

individual variability in cognitive, socio-emotional and neurological development (6-8). Therefore, GC cannot simply be presumed.' A couple of suggestions re this sentence. First, the final sentence 'GC cannot simply be presumed' doesn't quite reflect the line of argument in the preceding sentences – the evidence you give suggests that you're not just arguing that GC cannot be presumed, rather that evidence suggests many children and young people may have the competence to participate, and therefore we need means of assessing that? Also, in case of interest, this paper provides evidence that even younger children are presumed competent to participate in medical decisions - Children's ages of consent to non-urgent heart surgery: The views of two paediatric cardiology teams - Alderson - 2024 - Children & Society - Wiley Online Library

Thank you so much for drawing our attention to the work of Pamela Alderson. The first author has engaged with this extensively as part of making these amendments. We have added references to several of her papers in both the introduction and discussion.

Page 3 – lines 44-45: 'These practices are not typically followed in online studies, which tend to opt to seek parental consent for <16's.' This statement needs a citation to verify it.

This point has been amended and a citation added (i.e., Pearce & Buck, 2024).

Page 3 line 54 - page 4 line 10: This section is very much focused on concepts such as reliability and validity – 'representation and generalizability but can also impact the validity and reliability of a study' but these concepts relate primarily to quantitative rather than qualitative research. You might also consider children's right to participate in research, and in general the important knowledge lost from the most marginalised groups – see e.g. this paper which argues for the 'undeniable need to listen to children and adolescents as members of society; consider children's rights to actively participate, and voice their beliefs, opinions and experiences for methodological reasons, as it could enhance the quality and use of knowledge produced for the common good.' Children and adolescents' voices and the implications for ethical research - Hayda Alves, Lisa Gibbs, Katitza Marinkovic, Irma Brito, Payam Sheikhattari, 2022 (sagepub.com)

Many thanks for drawing our attention to this work. We have now made reference to it in the discussion section. We have also included it in the introduction as follows (pg.4):

It also potentially violates YP's rights to engage in research accorded to them in the United Nations Convention on the Rights of the Child as well as by the Children's Act of 1989 in England and Wales. Furthermore, consent has been highlighted by researchers and ethics committee members as a major challenge faced in conducting child and adolescent research (21).

Page 4 - lines 12-20: 'Although the option of GC has yet to become common practice within online research' – This sentence could be clearer - GC referes to 'Gillick Competence' rather than assessment of GC – i.e. you could say something like 'the option of assessing GC has yet to become common practice within online research'? And later 'an approach has been developed in Australia that allows <16s to consent to online research participation through assessing GC using a multiple choice test before signing the consent form.'

We have reflected on your comments, and have proposed the following changes to this section (pg.4):

Although the option of GC assessment to consent to research has yet to become common practice within online studies conducted in the UK, an approach has been developed in Australia that allows minors to consent to online research participation through GC, which is assessed using a multiple choice test which they complete before the consent form (22).

Methods

Page 4 – line 44-45: 'to assess comprehension' – please could you add comprehension of what? – e.g. 'to assess comprehension of the study information and the implications of participation'?

We agree with your comments and have added this, with additional context to relate it back to the facets of Gillick Competency (pg.5):

to assess comprehension of the study information (i.e. the purpose and process) and implications (i.e. risks and benefits) of taking part in the study.

Page 6 – lines 9-10: I was not clear how people were recruited to the quantitative part of the study – I think this sentence may be describing that but could be clearer - 'routed them to a separate Qualtrics survey from Project Care UK' – does the 'separate study' refer to this study? i.e. people were routed to answer the MCQs and answer questions about their interest in showing GC / using an online tool to show GC? I think my confusion relates to the fact that you only frame the qualitative work as a 'sub-study' and not the quantitative aspects of the pilot? Might it be better to frame the pilot as a 'mixed-methods sub-study' within the Project Care UK intervention evaluation?

This has been resolved now that we have removed the quantitative data from the manuscript, based on reviewer feedback.

Page 6 – line 19: 'After Qualtrics responses for bots' – I don't understand what this sentence means? Are there some missing words here? What does 'for bots' mean? e.g. (assuming I've understood correctly) you could say something like - 'After checking Qualitrics using RECAPTCHA to filter out any responses by bots (automated rather than human survey responses) …'?

Thanks for highlighting this, we have added some points to contextualise this section (pg.6):

Qualtrics responses were screened for potential bots (i.e. filtering out Qualtrics forms which scored ≤0.5 on the RECAPTCHA question as this indicates that the respondent might not be genuine).

Page 6 – line 20: 'Semi-structured interviews incorporating think-aloud techniques' –a citation is needed here for 'think-aloud techniques' – you provide one later but good to include it here too.

This has been added (i.e., Davies, 2018).

Results & Discussion:

The section on the quantitative results is quite brief – but I can't really see what could be added. The reporting of the qualitative findings is nicely detailed. My one key comment would be to include a bit more commentary on the recommendations for improvement (currently only reported in table 5). This could also be picked up on in the discussion.

Due to word count limitations, we have not substantially expanded on this, but we do believe that our wider work on the discussion section has addressed this at least to some extent.

Page 15 – lines 42-45: This section is really not clear what the 'qualitative sub-study' refers to – I had assumed you meant just the qualitative interviews – but this line 'However, both the main Project Care UK study and the qualitative sub-study were affected by fraudulent responses, and we used both automated and manual checks to discount likely bot responses (details on request).' i.e. I can't see how the qualitative interviews could have been affected by fraudulent responses / bots – so when you refer to the 'qualitative sub-study' are you including the survey / MCQs?

Apologies for the lack of clarity. We have now made amendments in both the methods section and the discussion section (limitations) to clarify, and also to address reviewer 2's feedback on a similar point.

Page 15 – lines 51-52: 'but we had just provided them with information and consented them to the sub-study'. Sorry this is a real bug-bear of mine and a common problem but researchers can't 'consent participants' – participants provide consent to take part in research – so suggest to reword e.g. 'But we had just provided them with information and they had consented to take part.'

Thanks for noticing. We have updated this in line with your comments.

Page 6 line 12: 'chance to register their interest to participant through' should read participate not participant.

Thanks for noticing. We have amended this.

Page 16 – lines 14-15: What is meant here by 'research settings' are you referring to more complex or higher risk research? If so perhaps make that clearer. And also what is meant by 'effectiveness' do you just mean acceptability and feasibility across diverse populations and research contexts? Or do you mean that testing 'effectiveness' is needed? If so effectiveness of what?

We believe that our extensive reworking of that section has resolved these issues. The conclusion now reads (pg.16):

In conclusion, our study provides preliminary evidence supporting the use of MCQs to assess GC in the context of low risk, anonymous, online mental health intervention research for YP aged <16 in the UK. By enabling YP to demonstrate their understanding and consent independently, this approach has the potential to expand the reach of research studies, the representativeness of the evidence-base, and the effectiveness of resultant interventions. However, further research is warranted to explore the feasibility and validity of this approach for GC assessment to consent to participate in research studies, including across diverse populations and types of research studies, while also ensuring that appropriate safeguards are in place to protect the rights and well-being of YP.

Reviewer: 2

A much-needed study in an under researched area, however, I do have some significant concerns and suggested revisions.

Thank you for your encouragement, it is much appreciated.

1: "The semi-structured interview was piloted with a young person (aged 16) to provide feedback, which resulted in changes to improve the clarity and flow of the interview schedule." It would be helpful to expand on what was changed and whether that was directly from feedback from the young person or from what was observed in the pilot interview by the researchers.

Thanks for pointing out this omission. We have expanded on our description of the piloting process as follows (pg.8):

The semi-structured interview was piloted with a YP (aged 16) to provide feedback, who advised the researchers to spend more time explaining what the research involved, which was subsequently incorporated into the interview schedule. No further changes were made at this stage to the flow of

the interview schedule as the pilot interview seemed to flow well. This pilot interview was not treated as generating data for the current study.

2: The MCQ development is a critical aspect of this study. It would be helpful to expand further on how they were developed initially from the previous research, including more information about the PPI conducted with young people (eg number of young people, ages, how many drafts they reviewed)

This is detailed in the method section as follows (pg.6):

The GC assessment MCQs were developed specifically for this study, based on prior research in Australia (23). Initially, MEL drafted four MCQs, each of which had four answer options, one of which was correct. The MCQs assessed (1) understanding of what the study involved, (2) why it was being done, (3) what the risks were of taking part and (4) the potential benefits of taking part (see Table 1). MEL shared the draft MCQs with a colleague who was chair of the Psychology department's ethics committee. The colleague provided feedback and suggestions regarding simplifications. MEL made these amendments, and then sought input from two young adults (undergraduate students), resulting in several further changes to the wording of the questions and answer options provided. The young adults then reviewed the changes and discussed and agreed refinements in a meeting with MEL.

3: I note a convergent mixed method approach. It would be helpful to understand the rationale for a convergent approach.

Thank you for highlighting this point. We have now removed the quantitative component of the study so it is no longer mixed methods.

4: There was a very large number of forms removed from analysis in data cleansing, some consideration about whether all of these were bots or some participants would be helpful. For example is it possible that some young people did not complete due to being unable to answer the MCQs?

We agree that this is possible. The data cleansing was a multi-step process. First, we filtered out forms based upon an automated fraud detection measure (e.g., RECAPTCHA). If participants scored ≤0.5 on the RECAPTCHA question, they were considered to be potential bots and filtered out as this indicates that a form might not be genuine. Next, those who were ineligible (i.e. those who self-reported that they were aged <13 or >18, and those unable to communicate in English) and incomplete (e.g., participants who did not complete or attempt the GC questions) forms from genuine participants were also removed. We then undertook manual checks based on existing guidance about ways to identify potentially fraudulent responses (see methods section additions). We have also updated our limitations section of the discussion to explicitly acknowledge this possibility.

5: It needs to be clearer whether the interviews were conducted with parents present or not and the rationale if it was with parents.

Included in the participants section, we state the following (pg.5):

YP and parent interviews were conducted independently, with data remaining unpaired even if a parent-child dyad participated.

6: Only 18 young people under 16 completed the survey, the vast majority identified as white british which is a significant limitation and I have to question the results with such a limited sample size.

We agree and on reflection, feel like it is better to remove the quantitative data and to focus on the qualitative data on acceptability.

7: Statistical analysis was conducted but there is no mention of this in the methodology

We have now removed the statistical components.

8: Further rationale needed around why the 16-18 year age group was included in the survey.

We have carefully considered this feedback, and on reflection, we have decided to remove the quantitative component of the study, and focus on the qualitative component.

9: Changing the interview due to confusion noticed is appropriate but suggests that the interview was not sufficiently piloted before being deployed.

Yes, we agree, and it was an important learning point for us as a team. We piloted it with one individual and it worked well, but piloting it again with another one or two individuals would have been helpful.

10: Objective 1 has not been met as no inference can be made about the proportion

We agree – this has now been removed entirely through removing the quantitative components.

11: Due to the concerns above I am not sure the conclusion is correct, it could also have been concluded that this was evidence that parental consent continues to be required and may be needed in those over 16 from the quantitative results.

Yes – we agree – and there is a huge amount of uncertainty around those findings given the issues with regard to sample size, so to be more confident a much larger, more robust quantitative study would be required.

12: The strength of this study is the qualitative work, maybe the study would be best reframed with emphasis on this and discussing how the interviews could inform a further review of how to consent young people under 16 (eg using a creative approaches such as videos to provide accessible information).

We agree and this was our planned way forwards - we have now explicitly included this in the discussion as follows (pg.15):

It is also important that information sheets for studies are provided in a range of formats, including audio and/or audio-visual formats to enable YP to engage with them. This kind of material could be co-created with YP to maximise its relevance and appropriateness.

VERSION 2 - REVIEW

**Reviewer**	**1**
**Name**	**Sutcliffe, Katy**
**Affiliation**	**University College London Institute of Education, Social Science**
**Date**	**12-Feb-2025**

COI

The authors have made comprehensive and appropriate revisions in response to the comments from me and the other reviewers.

One issue that I raised has not been addressed. I recommend that the authors consider revising the first sentence of the abstract: 'Providing digital mental health interventions online could expand access to help for young people, but requiring parental consent may be a barrier to participation.' This sentence implies that the paper is about young people consenting to receive a mental health intervention rather than consenting to take part in research. To avoid confusing readers, and to be consistent with the rest of the paper, a revised first sentence could read something like: ‘Requiring parental consent may be a barrier to research participation for young people.'

I recommend a revision to address this point, but will leave it for the authors to make that decision. I am happy for this paper to be published without reviewing it further.
